# Correction: Controlling barrier height and spectral responsivity of p–i–n based GeSn photodetectors *via* arsenic incorporation

**DOI:** 10.1039/d3ra90040a

**Published:** 2023-05-11

**Authors:** Mohamed A. Nawwar, Magdy S. Abo Ghazala, Lobna M. Sharaf El-Deen, Badawi Anis, Abdelhamid El-Shaer, Ahmed Mourtada Elseman, Mohamed M. Rashad, Abd El-hady B. Kashyout

**Affiliations:** a Physics Department, Faculty of Science, Menoufia University Menoufia Shebin El-Koom 32511 Egypt mohamed.nawwar@science.menofia.edu.eg; b Spectroscopy Department, Physics Research Institute, National Research Centre 33 El Bohouth St., Dokki Giza 12622 Egypt; c Physics Department, Faculty of Science, Kafrelsheikh University KafrelSheikh 33516 Egypt; d Electronic & Magnetic Materials Department, Advanced Materials Institute, Central Metallurgical Research & Development Institute (CMRDI) Helwan-Cairo 11421 Egypt; e Electronic Materials Department, Advanced Technology and New Materials Research Institute, City of Scientific Research and Technological Applications (SRTA-City) New Borg El-Arab City Alexandria 21943 Egypt akashyout@srtacity.sci.eg

## Abstract

Correction for ‘Controlling barrier height and spectral responsivity of p–i–n based GeSn photodetectors *via* arsenic incorporation’ by Mohamed A. Nawwar *et al.*, *RSC Adv.*, 2023, **13**, 9154–9167, https://doi.org/10.1039/D3RA00805C.

The authors regret that the author associations with affiliations were incorrectly shown in the original manuscript. The corrected list of affiliations is as shown above.

The Royal Society of Chemistry apologises for these errors and any consequent inconvenience to authors and readers.

## Supplementary Material

